# Supplementary material for: Risk-Based Mapping Tools for Surveillance and Control of the Invasive Mosquito Aedes albopictus in Switzerland
Source: Int J Environ Res Public Health. 2022 Mar 9;19(6):3220. doi: 10.3390/ijerph19063220 (PMC8955472; doi:10.3390/ijerph19063220)
Supplement: Supplementary file 1 [file ijerph-19-03220-s001.zip › Figure_S4.pdf]

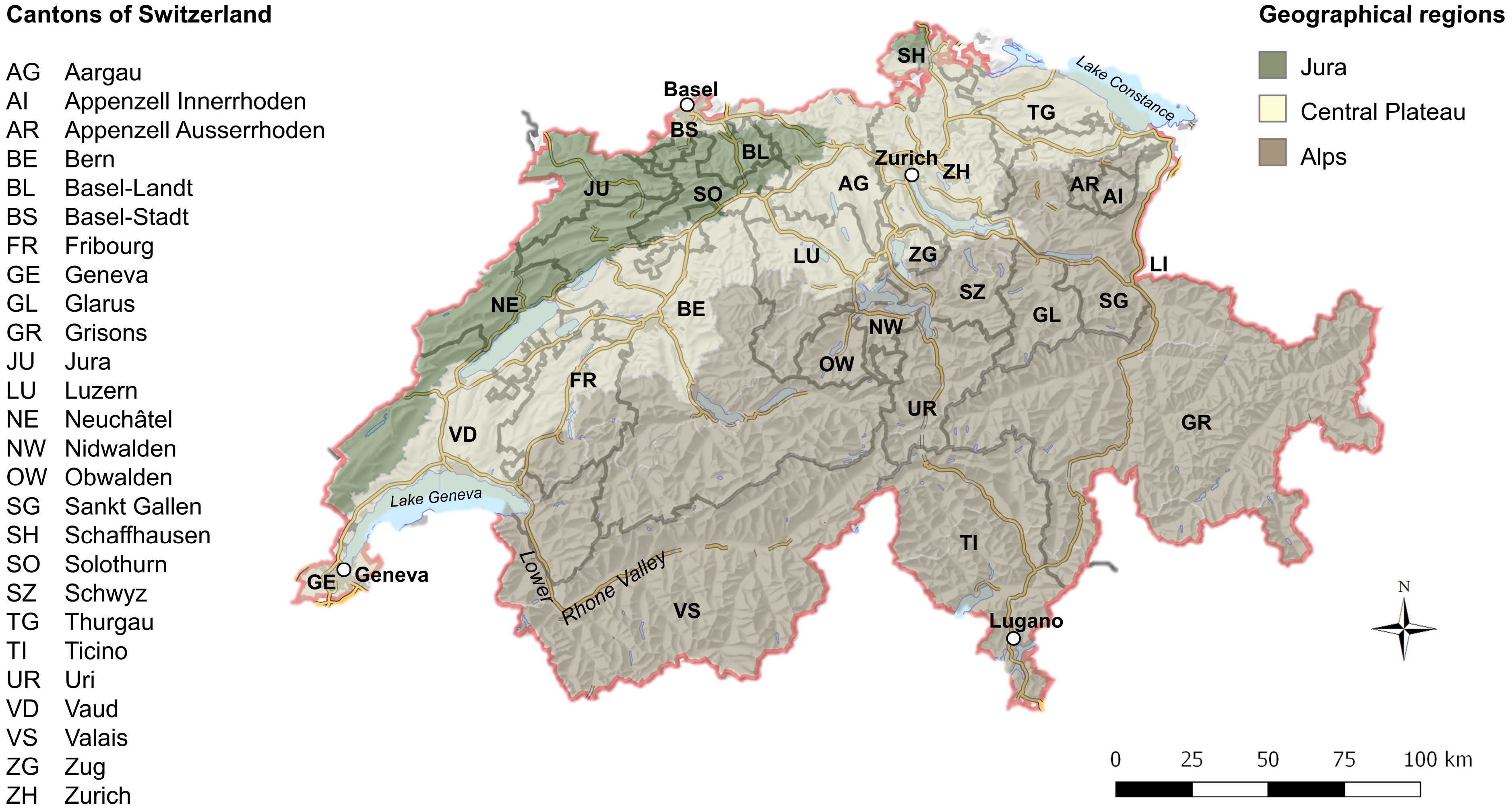

Figure S4. Map of Switzerland showing the three main geographical regions and the 26 cantons with their boundaries. Source of main map: Swiss Federal Office of Topography, modified in qGIS 3.0.3. Map of geographical regions by Romano 1246, distributed under a GNU Free Documentation License.
