# Supplementary material for: Risk-Based Mapping Tools for Surveillance and Control of the Invasive Mosquito Aedes albopictus in Switzerland
Source: Int J Environ Res Public Health. 2022 Mar 9;19(6):3220. doi: 10.3390/ijerph19063220 (PMC8955472; doi:10.3390/ijerph19063220)
Supplement: Supplementary file 1 [file ijerph-19-03220-s001.zip › Table_S1.pdf]

Table S1: Candidate predictors used for modelling *Ae. albopictus* habitat suitability

Category	Source	Predictor (Unit of Measure)	Abbreviation
Terrain morphology	DHM25 / 200m (Swiss Federal Office of Topography)	Altitude (m.a.s.l.)	elevation
		Land orientation (land direction with respect to the magnetic North)	aspect
		Slope (%)	slope
Land cover types	CORINE Land Cover 2012 (CLC-CH 2012)	Percentage of continuous urban fabric	clc111 (urban) land cover
		Percentage of discontinuous urban fabric	clc112 (urban) land cover
		Percentage of industrial or commercial units	clc121 (industrial) land cover
		Percentage of road and rail networks and associated land	clc122 (transport) land cover
		Percentage of port areas	clc123 (port) land cover
		Percentage of airports	clc124 (airport) land cover
		Percentage of mineral extraction sites	clc131 (extraction) land cover
		Percentage of dump sites	clc132 (dump) land cover
		Percentage of construction sites	clc133 (construction) land cover
		Percentage of green urban areas	clc141 (green) land cover
		Percentage of sport and leisure facilities	clc142 (leisure) land cover
		Percentage of non-irrigated arable land	clc211 (arable) land cover
		Percentage of vineyards	clc221 (vineyards) land cover
		Percentage of fruit trees and berry plantations	clc222 (plantation) land cover
		Percentage of pastures	clc231 (pasture) land cover
		Percentage of complex cultivation	clc242 (cultivation) land cover
		Percentage of land principally occupied by agriculture, with significant areas of natural vegetation	clc243 (agriculture) land cover
		Percentage of broad-leaved forest	clc311 (forest) land cover
		Percentage of coniferous forest	clc312 (forest) land cover
		Percentage of mixed forest	clc313 (forest) land cover
		Percentage of natural grassland	clc321 (grassland) land cover
		Percentage of moors and heathland	clc322 (moors) land cover
		Percentage of transitional woodland shrub	clc324 (shrub) land cover
		Percentage of beaches, dunes, and sand plains	clc331 (sand) land cover
		Percentage of bare rocks	clc332 (rocks) land cover
		Percentage of sparsely vegetated areas	clc333 (vegetated) land cover
		Percentage of burnt areas	clc334 (burnt) land cover
		Percentage of glaciers and perpetual snow	clc335 (glaciers) land cover
		Percentage of inland marshes	clc411 (marshes) land cover

Category	Source	Predictor (Unit of Measure)	Abbreviation
		Percentage of peatbogs	clc412 (peatbog) land cover
		Percentage of water courses	clc511 (water) land cover
		Percentage of water bodies	clc512 (water) land cover
Social factors	STATPOP2016 (GEOSTAT)	Total human population	human population
Meteorological factors	MeteoSwiss spatial climate datasets	5th percentile of the minimum temperature in the cold season (°C)	cold-s- Tmin p5
		25th percentile of the minimum temperature in the cold season (°C)	cold-s Tmin p25
		75th percentile of the minimum temperature in the cold season (°C)	cold-s Tmin p75
		95th percentile of the minimum temperature in the cold season (°C)	cold-s Tmin p95
		Average of the minimum temperature in the cold season (°C)	cold-s Tmin average
		5th percentile of the maximum temperature in the cold season (°C)	cold-s Tmax p5
		25th percentile of the maximum temperature in the cold season (°C)	cold-s Tmax p25
		75th percentile of the maximum temperature in the cold season (°C)	cold-s Tmax p75
		95th percentile of the maximum temperature in the cold season (°C)	cold-s Tmax p95
		Average of the maximum temperature in the cold season (°C)	cold-s Tmax average
		5th percentile of the mean temperature in the cold season (°C)	cold-s Tmean p5
		25th percentile of the mean temperature in the cold season (°C)	cold-s Tmean p25
		75th percentile of the mean temperature in the cold season (°C)	cold-s Tmean p75
		95th percentile of the mean temperature in the cold season (°C)	cold-s Tmean p95
		Average of the mean temperature in the cold season (°C)	cold-s Tmean average
		5th percentile of the minimum temperature in the warm season (°C)	warm-s Tmin p5
		25th percentile of the minimum temperature in the warm season (°C)	warm-s Tmin p25
		75th percentile of the minimum temperature in the warm season (°C)	warm-s Tmin p75
		95th percentile of the minimum temperature in the warm season (°C)	warm-s Tmin p95
		Average of the minimum temperature in the warm season (°C)	warm-s Tmin average
		5th percentile of the maximum temperature in the warm season (°C)	warm-s Tmax p5
		25th percentile of the maximum temperature in the warm season (°C)	warm-s Tmax p25
		75th percentile of the maximum temperature in the warm season (°C)	warm-s Tmax p75
		95th percentile of the maximum temperature in the warm season (°C)	warm-s Tmax p95
		Average of the maximum temperature in the warm season (°C)	warm-s Tmax average
		5th percentile of the mean temperature in the warm season (°C)	warm-s Tmean p5
		25th percentile of the mean temperature in the warm season (°C)	warm-s Tmean p25
		75th percentile of the mean temperature in the warm season (°C)	warm-s Tmean p75
		95th percentile of the mean temperature in the warm season (°C)	warm-s Tmean p95
		Average of the mean temperature in the warm season. (°C)	warm-s Tmean average
		75th percentile of the precipitations in the cold season (mm)	cold-s RAIN p75

Category	Source	Predictor (Unit of Measure)	Abbreviation
		95th percentile of the precipitations in the cold season (mm)	cold-s RAIN p95
		Average of precipitations in the cold season (mm)	cold-s RAIN average
		75th percentile of the precipitations in the warm season (mm)	warm-s RAIN p75
		95th percentile of the precipitations in the warm season (mm)	warm-s RAIN p95
		Average of precipitations in the warm season (mm)	warm-s RAIN average
		Maximum Tmin (daily minimum temperature) observed during the two-week period of the year with the lowest maximum Tmin (°C)	Tmin minimum biweekly maximum
		Average Tmin (daily minimum temperature) observed during the two-week period of the year with the lowest average Tmin (°C)	Tmin minimum biweekly average
		Minimum Tmax (daily maximum temperature) observed during the two-week period of the year with the highest minimum Tmax (°C)	Tmax maximum biweekly minimum
		Average Tmax (daily maximum temperature) observed during the two-week period of the year with the highest average Tmax (°C)	Tmax maximum biweekly average
		Average precipitation observed during the two-week period of the warm season with the highest average precipitations (mm)	warm-s RAIN maximum biweekly average
		Average precipitation observed during the two-week period of the warm season with the lowest average precipitation (mm)	warm-s RAIN minimum biweekly average
Social factors	OSRM, OSM	Road-based distance in minutes of travel by car from the nearest cell established in the previous year (min)	car distance to establishment
